# Natural Microbiota of Dogs and Cats as a Source and Vector of Resistance Genes—Clinical Significance

**DOI:** 10.3390/ijms26167717

**Published:** 2025-08-09

**Authors:** Iga Horodyska, Patrycja Kasperska, Kacper Michalski, Joanna Bubak, Izabela Herman, Marta Miszczak

**Affiliations:** 1 EZA Student Science Club, Department of Epizootiology and Clinic of Birds and Exotic Animals, Faculty of Veterinary Medicine, Wrocław University of Environmental and Life Sciences, 31 Norwida St., 50-375 Wrocław, Poland; 120498@student.upwr.edu.pl (I.H.); 120488@student.upwr.edu.pl (P.K.); 120443@student.upwr.edu.pl (K.M.); 2 Department of Pathology, Division of Pathomorphology and Veterinary Forensics, Faculty of Veterinary Medicine, Wrocław University of Environmental and Life Sciences, 31 Norwida St., 50-375 Wrocław, Poland; joanna.bubak@upwr.edu.pl; 3 Vetspec Companion Animal Healthcare Centre, Odrzanska 9, 55-003 Czernica, Poland; izabela.herman@o2.pl; 4 Department of Epizootiology and Clinic of Birds and Exotic Animals, Division of Infectious Diseases and Veterinary Administration, Faculty of Veterinary Medicine, Wrocław University of Environmental and Life Sciences, Grunwaldzki Sq.45, 50-366 Wrocław, Poland

**Keywords:** natural microbiota, resistance genes, vectors, drug resistance, dogs, cats, surgical procedures, one health, antimicrobial resistance, endogenous infections

## Abstract

Antimicrobial resistance (AMR) presents a growing global threat, driven by widespread antibiotic misuse across human and veterinary medicine. Companion animals, particularly dogs and cats, harbor complex natural microbiota—including skin, mucosal, and gastrointestinal communities—that are essential to their health yet also serve as reservoirs of antibiotic resistance genes (ARGs). These ARGs can spread through horizontal gene transfer (HGT), especially during bacterial imbalances such as endogenous infections or surgical interventions, increasing the risk of difficult-to-treat infections. Documented zoonotic and anthroponotic transmissions of resistant strains such as MRSA, MRSP, and ESBL-producing *E. coli* highlight the bidirectional nature of ARG flow between animals and humans. This underscores the critical importance of the One Health approach, which promotes interdisciplinary collaboration to monitor, understand, and combat AMR across the human–animal-environment interface. Key mechanisms of ARG dissemination, the role of companion animal microbiota, and real-world examples of resistance transfer between species illustrate the complexity and urgency of addressing AMR. Targeted surveillance, rational antibiotic use, and public awareness are essential to preserving antimicrobial efficacy and safeguarding both human and animal populations.

## 1. Introduction

Upon the introduction of antibiotics, it was assumed that the evolution of AMR was unlikely. This perspective was based on the assumption that the frequency of mutations leading to resistance in bacteria was negligible [1]. This has proven false, as bacteria exposed to various chemicals have adapted by developing resistance to antibiotics. AMR in animals now undermines effective treatment in livestock, companion, exotic, and wild species, leading to therapy failure. Increased mortality and serious welfare concerns as once-reliable drugs lose their efficacy [2,3]. This is where One Health’s approach is critical. Its approach is to sustainably balance and optimize the health of people, animals and ecosystems, which operates on the idea of collaboration across sectors and disciplines [4].

Nowadays, mitigating the risk of further evolution of resistance involves several strategies. Some of the most important ones include: identifying bacteria and their susceptibility to different substances, using those substances in standardized therapeutic schemes, incorporating systems such as the Rational Antibiotic Use System (RAUS) [5,6,7].

Bacteria exhibit various independent mechanisms of AMR, including—but not limited to—alterations in cell envelope permeability, active efflux of antibiotics (the so-called efflux system), enzymatic inactivation, and modification of antibiotic targets. These are just a few of the most important mechanisms with which microorganisms defend against toxic substances [8]. Those mechanisms can come from two types of AMR: intrinsic and acquired. Intrinsic resistance is limited by the structural and functional features of the bacteria and is developed using naturally occurring mechanisms. Acquired resistance results from changes in the bacterial genome, either from mutations in antibiotic-targeted genes or from acquisition of exogenous DNA [9]. Both of these pathways lead to the development or possession of ARGs. The origin of ARGs is not clear, but one of the explanations for their existence is based on the idea of development from other functional genes or even from non-coding sequences—the intrinsic pathway. This idea is supported by the fact that antibiotic resistance exists in totally uninhibited or thinly populated areas, where the concentration of antibiotics is minimal [10,11]. What is important to understand is that any bacteria can develop resistance—regardless of whether it is considered harmful, neutral, or beneficial to the host organism [12]. With this in mind, the importance of RAUS policy becomes clear. Simply using antibiotics can result in the development of resistance in bacteria that are considered natural, ones that are part of our and our animal’s microbiome [13]. This is very important, as endogenous bacteria can become resistant to antibiotics, and if an opportunistic infection were to happen, it would cause further diagnostic and therapeutic challenges, for example, in post-surgery patients suffering from endogenous skin microbiota infection or in patients that need immunosuppressant therapy. Considering the improper use of antibiotics, such as choosing wrong substances, forgetting to administer them or not administering them for a long enough period, we can expect the risk of bacteria developing resistance to increase [14]. Additionally, opportunistic bacteria, which can be a part of the body’s microbiota, can become pathogenic under the right conditions or help the invading, pathogenic bacteria to survive and replicate [15]. It means that there is always a risk of localized resistance development, as every bacteria, even resident ones, can take advantage of different mechanisms for transferring genes between them.

The most important defense against AMR and the spread of ARGs is the knowledge about them. Research allows us to understand and identify critical factors affecting the bacteria and the transfer rates. Having them identified opens up new ways for fighting them. This is why research on ARGs is so valuable to the public health sector. While the research should be conducted on a very big scale, we believe that focusing on domestic and livestock animals should be a priority. The reasoning behind this approach is that we already know that the transfer occurs between aforementioned populations and humans. However, we do not have a list of all of the ARGs taking part in this process or the rates of these transfers. Combined with the increase in animals per household and adopting dogs or cats in place of having children, it is more likely that new ARGs will emerge and the rates of transmission of them will increase [16].

## 2. Microbiota

**Figure 1:**
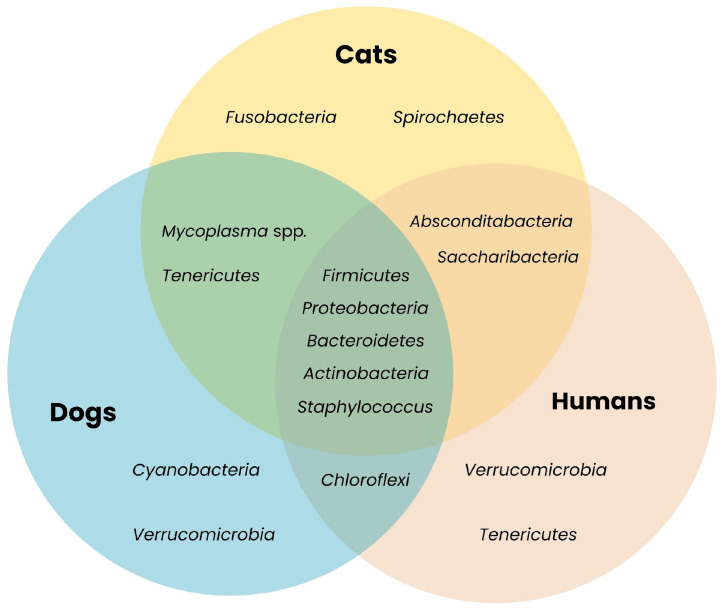
Shared bacterial taxa across companion animals and humans. The Venn diagram illustrates overlapping bacterial phyla present in microbiota of different species. The presence of shared phyla such as *Firmicutes*, *Bacteroidetes*, *Actinobacteria* and *Proteobacteria* highlights their potential role as ARGs reservoirs [18,19,20,22,23,24,25,26,27,28,29,30,31,32].

**Figure 2:**
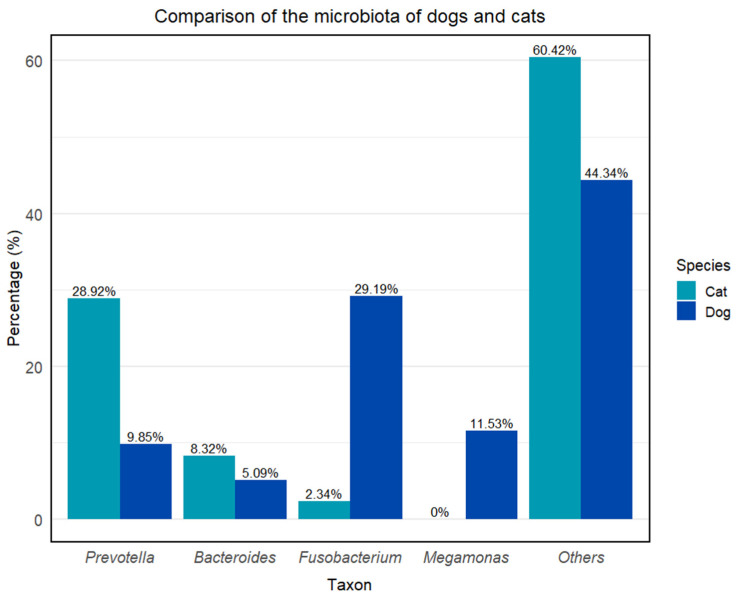
Comparison of the microbiota of dogs and cats. Others (dog): *Alloprevotella*—7.54%, *Haemophilus*—4.04%, *Anaerobiospirillum—*3.63%, *Helicobacter*—3.01%, *Megasphaera*—2.65%, *Peptoclostridium*—1.63%, *Phascolarctobacterium*—1.57%, *Streptococcus*—1.22% + no data available; others (cat): *Romboutsia*—5.28%, *Clostridium sensu stricto*—5.32% + no data available [33,34].

Microbiota is defined as the range of microorganisms that may be commensal, mutualistic, or pathogenic found in and on all multicellular organisms [17]. These include bacteria, archaea, protists, fungi, and viruses, but this article will focus mainly on bacteria. It has been proven that it plays key roles in several metabolic, nutritional, physiological, and immunological processes [18]. Depending on the localized regions, microbiota can be classified into oral, gut, respiratory, skin, and urogenital, among many other emerging niches such as biliary tract, central nervous system, etc. [19]. Not only does microbiota vary between body sites, but it also differs depending on species, geographic region, age, sex, diet, and other individual specific factors [18,20]. A core resistome exists across diverse animal hosts: bacterial phyla *Firmicutes* and *Bacteroidetes* are consistently dominant in dogs, cats and humans, indicating shared potential reservoirs for ARGs [19]. *Proteobacteria* and *Actinobacteria* also overlap among these groups, highlighting convergent microbial niches despite anatomical differences [18,19] (Figure 1 and Figure 2).

Under clinical conditions the microbiological balance can undergo significant changes, leading to dysbiosis and secondary complications. After surgical procedures, especially those involving the gastrointestinal tract, a decrease in the diversity of the intestinal microbiota is often observed [21]. For example, in dogs after surgery such as hemilaminectomy, an increase in the dysbiosis index (DI) was found, which indicates a disruption of the microbial balance [21]. In addition, surgical procedures contribute to an increase in the number of potentially pathogenic genera of bacteria, such as *Escherichia*, *Enterococcus* or *Pseudomonas*. The use of antibiotics in the perioperative period may disturb the balance of the microbiota, leading to a decrease in the number of bacteria sensitive to these drugs and an increase in the number of resistant bacteria [21].

**Figure 3:**
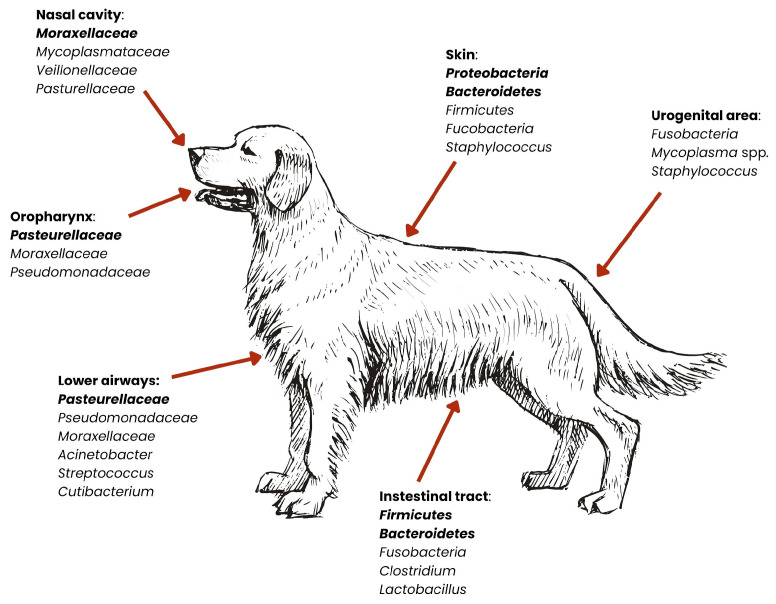
Distribution of dominant bacterial groups across anatomical niches in dogs. Relative abundances of bacterial phyla vary by body site. Dominant microbiota are marked in bold [27,29,36,37,38,39,40,41].

**Figure 4:**
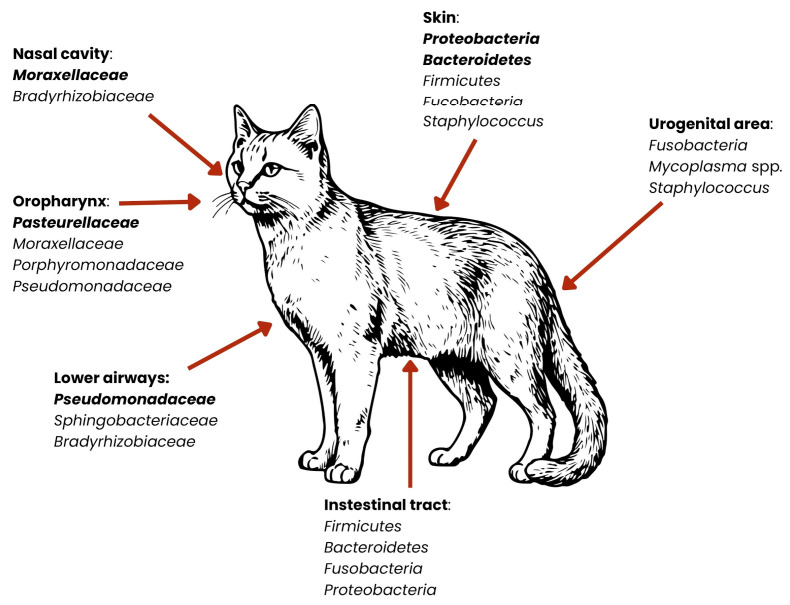
Distribution of dominant bacterial groups across anatomical niches in cats. Relative abundances of bacterial phyla vary by body site. Dominant microorganisms are marked in bold [27,30,36,37,38,39,40,41].

Despite the differences between animal species in the context of the microbiota of the organism, the greatest attention will be paid to dogs and cats, due to their closest contact with humans. Therefore, according to the One Health concept, they will be the source of resistance genes that can be passed on to humans [35]. Distinct body sites harbor characteristic communities of microorganisms. The schemes below present the subtle differences between dogs’ and cats’ microbiomes (Figure 3 and Figure 4).

The skin of dogs and cats is inhabited by many types of bacteria that help protect against pathogens and support the skin’s protective functions. A study by Whittle et al. (2024), based on skin metagenomics, indicates that the skin of companion animals is dominated by the phyla *Proteobacteria*, *Bacteroidota*, and *Actinobacteria*, and they constitute about 85% of the entire skin microbiota [38]. The remaining pool of bacteria includes the phyla *Firmicutes* and *Fusobacteriota* (1–7%) [38]. The composition of the skin microbiota, divided into specific bacterial species, is presented in Table 1. Although the bacterial composition of the skin is very similar in both dogs and cats, there are visible differences in the amounts of individual phyla in the entire microbiome—in dogs, *Proteobacteria* occur at a level of 36–40% of the entire microbiome composition, while in cats the same bacteria are present in approximately 46% of the entire skin microbiota [38,39]. In addition, in dogs, the phenomenon of the “individual microbiome” is observed, meaning that individual differences in the composition of the microbiota are visible [42]. In cats, on the other hand, it is the site of sampling (and thus the specific body area) that influences the differences in the structure of the bacterial community, rather than individual differences [39].

Research on the reproductive microbiota in dogs has accelerated with the emergence of new, culture-independent microbiota research techniques [43]. Studies indicate that the microbiome of the reproductive system is characterized by great diversity, and primarily differs depending on the sex of the animal [43]. In bitches, the most common are the phylum *Fusobacteriota* and the genera *Ralstonia*, *Hydrotalea*, and *Mycoplasma*, while in males, phyla such as *Proteobacteria*, *Firmicutes* and *Actinobacteria* (isolated from the second and third fractions of semen) dominate [43]. Bacteria inhabiting the reproductive system are crucial for its health, supporting immunity, preventing infections, and in females, supporting the proper course of pregnancy [43]. The feline reproductive system microbiota is not as well understood as the canine microbiota, but is currently being studied using the 16S sequencing method at the phylum level. A study by Banchi et al. (2024) shows that the microbiota of this body system is dominated by the phyla *Proteobacteria*, *Firmicutes*, *Bacteroidota*, and *Actinobacteria* [44].

Another area where natural microbiota occur is the respiratory system. The misconception that described the respiratory tract as a sterile area of the body has been disproved, thanks to, among other things, advances in the field of metagenomics, and it is now known that the respiratory system is occupied by naturally occurring microorganisms [41]. In both dogs and cats, the dominant bacteria in the nasal cavity are the phyla: *Bacteroidota* and *Firmicutes*, as well as the phylum *Proteobacteria*, which also occur in the lower respiratory tract [45]. In dogs, the natural microbiota in the lungs is also bacteria from the genera *Cutibacterium*, *Streptococcus*, *Acinetobacter*, and *Pseudomonas* [41]. It should be mentioned that the respiratory microbiota varies depending on the dog breed. Studies indicate that the bacterial profile is different for brachycephalic dogs compared to dolichocephalic or mesocephalic dogs, as well as compared to cats [46,47] (Table 2). There are no available sources that show such differences among cat breeds [38].

Bacteria residing in the oral cavity of dogs and cats are particularly significant in the context of zoonotic disease transmission, largely due to the close physical contact these animals have with humans—with many owners allowing face-licking or even kissing their pets [48]. This microbiota is a complex community of microorganisms that play a key role in maintaining oral health, as well as influencing the overall health of the animal. According to research conducted by Ruparell et al. (2020), microbiota vary within niches in the oral cavity, with supragingival plaque microbiota being the most diverse [36]. The main bacterial phyla throughout the oral cavity are *Proteobacteria*, *Bacteroidota* and *Firmicutes* (in varying proportions in supragingival plaque, buccal mucosa and dorsum of the tongue, and saliva). Furthermore, higher amounts of *Actinobacteria* and *Saccharibacteria* were found in supragingival plaque than in other parts of the oral cavity [36]. Studies show that the composition of the microbiota is similar among dogs and cats (phylum *Proteobacteria* and genus *Bacteroides* are dominant), but not only are their numbers within the bacterial species different, but there are also differences observed within different dog and cat breeds [37]. A study conducted by Mei et al. (2024), taking into account three dog breeds (Teddy, Maltese, and Golden Retriever) and three cat breeds (Chinese garden cat, British shorthair, and Ragdoll) showed significant differences in the number of microorganisms [37]. The main genera of bacteria in the saliva of Maltese and Teddy are *Porphyromonas* and *Moraxella*, while the dominant genera of bacteria in the saliva of Golden Retrievers are *Neisseria* and *Streptococcus*. In cats, differences were also evident—the salivary microbiota of British shorthair and Ragdoll cats were closer together with the presence of genera *Porphyromonas* and *Moraxella*, whereas in Chinese garden cats, the genera *Porphyromonas* and *Fusobacterium* were observed [37]. In the case of oral microbiota, current research does not provide specific bacterial species [36]. In cats, the bacteria are described as “feline oral taxon”—suggesting that they have not been assigned to known species [49].

The gut microbiota of dogs and cats is one of the most important metabolic organs, where bacteria play an important role in digestion and metabolism [50]. It also synthesizes vitamins and support the immune system, defending the body against intestinal pathogens [51]. The gut microbiota is also one of the most important components of ARGs dissemination—bacteria such as *Gammaproteobacteria* and *Enterobacteriaceae* have been shown as one of the key drivers of said dissemination [45,46]. The composition of the intestinal microbiota of these animals is diverse, but it is dominated by four main phyla of bacteria: *Firmicutes*, *Bacteroidetes*, *Proteobacteria* and *Actinomycetota* [40]. In dogs, according to You and Kim (2021), the main components of the microbiota are the genera *Fusobacterium*, *Clostridium*, *Bacteroides* and *Lactobacillus* [29]. In cats, however, the microbiota is also rich in bacteria from the phyla *Firmicutes* and *Bacteroidetes*, but the phyla *Fusobacteriota* and *Proteobacteria* are more common [30]. A study by Rojas et al. (2024) found that the gut microbiota of dogs is primarily composed of 23 bacterial species, which constitute an average of 75% of the total microbiota [52]. Among these, *Collinsella intestinalis*, *Megamonas funiformis*, *Peptacetobacter hiranonis*, *Prevotella copri*, *Turicibacter sanguinis*, and *Holdemanella biformis* are common [52]. They are particularly prevalent in dogs fed dry food. In contrast, dogs fed raw meat have a higher abundance of species such as *Bacteroides vulgatus*, *Caballeronia sordicola*, and *Enterococcus faecium* [52]. The study results indicate that diet has a significant impact on the composition of the gut microbiota in dogs, which may have further consequences for their health and digestion. Research conducted by Langon (2023) identified bacterial species that are found in cats but not in dogs [53]. These include *Blautia wexlerae*, *Collinsella aerofaciens*, *Flavonifractor plautii*, *Megasphaera elsdenii*, *Ruminococcaceae bacterium D16*, *Fusicatenibacter saccharivorans*, and *Bifidobacterium pullorum* [53].

## 3. Mechanism of Bacterial Resistance Transfer

**Figure 5:**
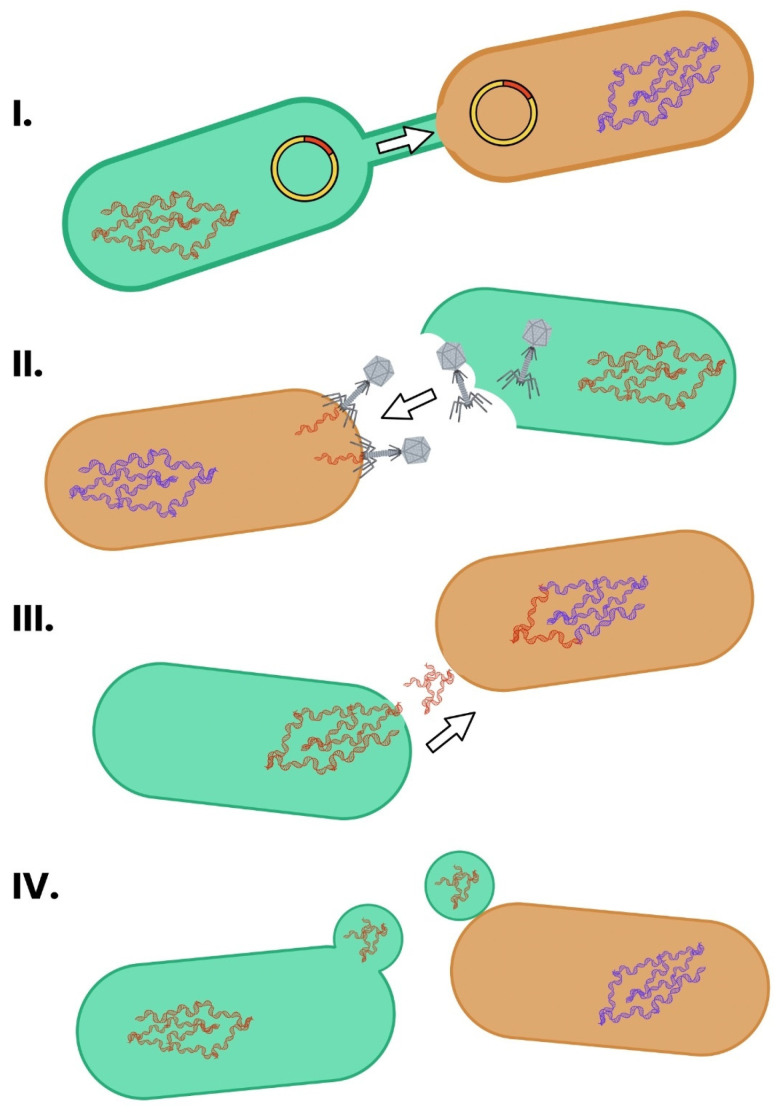
The main mechanisms of HGT in bacteria. The schematic depicts generic bacterial cells with specific structures that take part in the HGT. HGT occurs primarily through conjugation, transformation, and transduction. (**I**) Conjugation involves direct cell-to-cell contact and transfer of plasmids via a pilus. (**II**) Transduction occurs when bacteriophages transfer bacterial DNA between cells. (**III**) Transformation is the uptake of free DNA from the environment. (**IV**) Outer membrane vehicles, which are able to transport DNA, can fuse with recipient cells. These processes enable the rapid dissemination of ARGs across species and environments [57,58,61,62].

Understanding how ARGs are exchanged between bacteria is central to managing the spread of multidrug-resistant organisms in animal environments. The animal microbiome functions as both a source and vector of resistance genes, with HGT acting as the primary route for dissemination [54]. The three principal mechanisms of HGT—conjugation, transduction, and transformation—operate in conjunction with mobile genetic elements (MGEs) like plasmids, integrons, and transposons to enable the broad and rapid exchange of resistance determinants [54,55,56]. Conjugation involves direct cell–cell transfer of plasmid DNA via a type IV secretion pilus [57]. Transformation occurs when bacteria uptake free DNA fragments released into the environment and integrate them by homologous recombination [58]. Transduction is mediated by bacteriophages that mistakenly package host DNA (including ARGs) and inject it into new bacterial hosts during infection cycles [59]. These mechanisms collectively accelerate the spread of resistance determinants across species and ecological niches [60] (Figure 5).

### 3.1. Conjugation

Conjugation is the most significant mechanism for the horizontal spread of ARGs in bacterial communities, particularly in dense, diverse environments such as the gut microbiota of animals. This process involves the direct transfer of DNA between a donor and a recipient cell, mediated by a conjugative plasmid or integrative conjugative element [55]. A multiprotein complex forms between two cells, and a unidirectional transfer begins [63]. Plasmids, which are elements that autonomously replicate within the bacterial cell and mediate conjugation, often belong to broad-host-range incompatibility groups, such as IncP or IncN, allowing them to move freely between different species and even phyla of bacteria [55,64]. The process is initiated when the donor’s relaxosome complex recognizes and nicks the origin of transfer (oriT) on the plasmid. A single DNA strand is transferred through the type IV secretion system into the recipient, where it is recircularized and replicated [55]. Plasmids that encode multiple resistance determinants—often in the form of integrons or composite transposons—enable the co-transfer of multiple ARGs in a single conjugative event [55,65,66].

Recent studies have revealed new factors that modulate conjugation in microbial communities. For example, conjugative transfer can be inhibited by specific small molecules: unsaturated and alkynoic fatty acids (e.g., 2-hexadecynoic acid) are known to bind the VirB11-like ATPase (e.g., TrwD) of conjugative plasmids, displacing palmitic acid in the membrane and blocking its ATPase activity [67]. These compounds (called COINs, conjugation inhibitors) effectively reduce plasmid transfer without killing cells. In a related vein, Ott et al. (2024) demonstrated that diet-derived short-chain fatty acids (SCFAs)—such as acetate, propionate, and butyrate—can dramatically suppress conjugation in gut-like conditions [68]. In vitro and ex vivo (chicken cecal explant) experiments showed that millimolar SCFA concentrations almost completely blocked plasmid mating, significantly reducing transconjugant formation for plasmids of diverse incompatibility types. Because SCFAs are natural fermentation products in animal guts, this finding suggests that microbiome or diet factors can influence the rate of HGT in situ, adding a new layer to how conjugation is regulated in host environments [68]. Conjugation remains a powerful mechanism for ARG spread, but these insights into its modulation point to potential intervention strategies: blocking the transfer machinery or altering host metabolites could slow the horizontal dissemination of resistance.

### 3.2. Transduction

Transduction involves the accidental packaging and transfer of host DNA, including ARGs, by bacteriophages [69]. In generalized transduction, lytic phages infect a bacterial cell and package fragments of the host genome instead of phage DNA [69]. These defective particles can then inject host DNA into new bacterial cells, where it may recombine with the chromosome or be maintained episomally [69]. In specialized transduction, temperate phages that integrate into the bacterial genome excise incorrectly during induction, bringing adjacent host genes with them [70]. Although transduction events are generally less frequent than conjugation, their potential to shuttle resistance genes across species barriers is significant, especially in environments with high phage-to-bacteria ratios like the gut [56,69]. Phages may also act as reservoirs of resistance genes, contributing to the long-term persistence of ARGs in microbial communities [66]. They have a longer persistence than bacteria, thanks to their ability to survive in much harsher environments than them (acid and alkaline conditions, high temperatures, UV irradiation and chlorination resistance). However, in clinical settings, where most of the bacteria reside in biofilms, phages lose their battle against bacteria. Biofilms are much less susceptible to phage penetration, thanks to a broad spectrum of defense mechanisms (thickness, metabolic state of bacteria, diffusion inhibition, bacterial composition) [63]. Three different transduction pathways should be considered: generalized, specialized and lateral. The first one is based on the erroneous packaging—phages mispackage random segments of bacterial DNA instead of phage DNA. The second one occurs when phages incorrectly extract their DNA (together with host DNA). The third one is based on the naturally occuring phage transduction (bidirectional phage replication in situ), leading to DNA encapsidation prior to excision, and replication of long fragments of adjacent bacterial genes [63].

### 3.3. Transformation

Natural transformation is the process by which bacteria take up free DNA from their environment and incorporate it into their genomes [69]. This mechanism depends on the development of competence, a transient physiological state regulated by cell density, stress, and environmental cues, does not depend on MGEs [54,63]. Competent cells express membrane-bound DNA uptake complexes that bind and internalize double-stranded DNA, followed by degradation of one strand and integration of the other through homologous recombination [69]. While transformation is species-specific and limited by the requirement for sequence homology, it plays an important role in the spread of resistance among closely related organisms [69]. Biofilms, which are abundant in animal microbiomes, enhance transformation by providing high cell density and abundant extracellular DNA [65]. This can lead to higher upregulation in competence genes in *Staphylococcus aureus* and *Streptococcus pneumoniae* in biofilms rather than in broth-grown bacteria [63]. Furthermore, exposure to subinhibitory antibiotic concentrations can promote competence development and transformation efficiency [54].

Structural studies have recently filled in details of the DNA uptake machinery in Gram-positive species. For instance, in *Streptococcus pneumoniae* an extracellular pilus (composed of ComGC and related proteins) captures environmental DNA and retracts it towards the cell. The DNA-binding protein ComEA then binds this DNA, directing it to the transmembrane channel protein ComEC, which transports single-stranded DNA into the cytoplasm. The ComFA ATPase provides the energy for translocation, and nucleases, such as EndA cleave one strand to complete uptake. Once inside, single-stranded binding proteins and RecA facilitate recombination with the host chromosome. These detailed models (based on crystallography and genetics) illuminate how transformation is achieved at the molecular level in pathogens, although species vary in the specific proteins used [71].

### 3.4. Transposons

**Figure 6:**
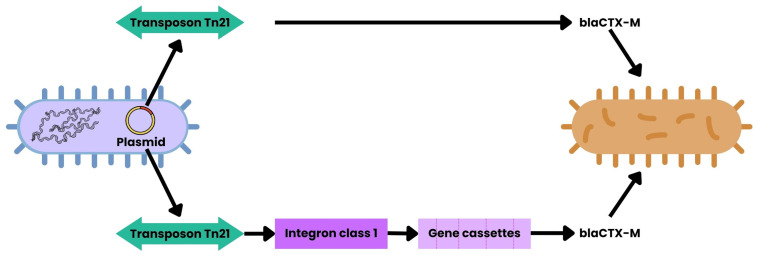
HGT mediated by MGEs. This schematic depicts generic bacterial cells. Plasmids may carry transposons, such as Tn21, which contain class 1 integrons that capture gene cassettes encoding ARGs (e.g., *aadA*, *dfrA*, *bla_CTX-M_*). Additionally, Tn21 can directly mobilize ARGs via insertion sequences. These elements are inserted into bacterial genomes, while bacteriophages enable ARGs transduction between species [55,56].

Transposons are mobile DNA elements that can move from one genomic location to another, often carrying resistance genes with them [55]. They typically encode a transposase that mediates their excision and reinsertion into new sites, whether on the same molecule or on different replicons [56]. Composite transposons, like Tn10 and Tn5, flank resistance genes with insertion sequences (IS elements), while more complex structures, such as Tn21 contain integrons and mercury resistance operons in addition to ARGs [54,55]. Some transposons are conjugative, meaning they contain both transposition and conjugation machinery, which greatly enhances their ability to disseminate resistance genes within and across bacterial populations [54]. MGEs function in a sequential cascade: plasmids harbor transposons (e.g., Tn21) that carry class 1 integrons with arrays of gene cassettes (e.g., *aadA*, *dfrA*) before integrating into recipient genomes [55]. Insertion sequences like IS*Ecp*1 can mobilize *bla*_CTX-M_ into new loci, while bacteriophages perform generalized and specialized transduction of ARGs across taxonomic boundaries [56] (Figure 6). These transposons often reside on plasmids, where they can integrate into the host genome or jump between plasmids, creating a mosaic of resistance loci.

### 3.5. Integrons

Integrons are genetic platforms that capture, integrate, and express gene cassettes—many of which encode resistance to antibiotics [72]. The core components of an integron include the integrase gene (*intI*), a recombination site (*attI*), and a promoter that drives expression of the inserted genes [55]. Class 1 integrons are particularly common in clinical and agricultural settings and are frequently found embedded within transposons, such as Tn21 [55,72]. Integrons do not themselves mobilize but rely on transposons and plasmids for movement [56]. In animal-associated bacteria, especially within the gut, integrons act as accumulators of resistance genes, facilitating rapid adaptation under antibiotic pressure [73]. Their cassette arrays can expand over time, with newer cassettes inserted upstream, thereby gaining stronger expression under the integron’s promoter. This mechanism allows bacteria to respond dynamically to changes in selective pressure, accumulating and reshuffling resistance genes as needed [56,73].

Recent experimental evolution work has directly demonstrated the power of integrons to accelerate resistance evolution. Souque et al. [73] engineered *Pseudomonas aeruginosa* with a custom class-1 integron carrying three different antibiotic-resistance cassettes. Under stepwise antibiotic selection, bacteria with an active integrase rapidly duplicated the needed resistance gene and jettisoned redundant cassettes, dramatically increasing survival. In contrast, integrase-deficient mutants adapted much more slowly. This confirms that integrase-mediated shuffling (“resistance on demand”) allowed a rapid, targeted boost in expression of the beneficial gene without apparent off-target cost [73].

The result of a combination of aforementioned mechanisms and environmental factors is a dynamic network. In this network resistance genes are not only maintained but actively disseminated through bacterial populations, sometimes spilling over into pathogens of clinical significance [55,56,66].

### 3.6. Outer Membrane Vesicles

In addition to the classical HGT routes, outer membrane vesicles (OMVs) have emerged as novel vehicles for gene transfer in Gram-negative bacteria. OMVs are nano-scale spheres released from the outer membrane that carry lipids, proteins, and nucleic acids. Crucially, DNA packaged inside OMVs is protected from extracellular DNases. Multiple studies have shown that OMVs can fuse with recipient cells and deliver genetic material. For example, OMVs from Acinetobacter baumannii and Escherichia coli have been shown to carry and spread extended-spectrum β-lactamase genes (e.g., *bla*_CTX-M_-15) and carbapenemase genes (e.g., bla_NDM-1) [61]. In one striking case, Hua et al. [62] demonstrated that OMVs produced by a carbapenem-resistant hypervirulent Klebsiella pneumoniae simultaneously packaged both a carbapenemase gene (bla_KPC) and virulence genes. When these OMVs fused with nonpathogenic K. pneumoniae recipients, the transformants acquired both resistance and virulence traits—effectively creating new carbapenem-resistant hypervirulent strains [62]. These findings underscore that OMVs can co-transfer ARGs and other functions between bacteria, representing an additional layer in the HGT network.

## 4. Transmission of ARGs Across Hosts

One of the most important elements in understanding the spread of AMRs is studying the mobile ARGs and their pathways. A study by Hu et al. (2016) found the following: the HGT frequency of mobile ARGs in bacteria was the highest in animal-associated bacteria (AAB; bacteria isolated from animal hosts), while second place was taken by human-associated bacteria (HAB; bacteria isolated from humans) [74]; the mobile ARGs were exchanged most frequently between AAB and HAB; and in both AAB and HAB, approximately 35% of the bacteria carried mobile ARGs [74]. These findings indicate that the mobile resistome is a very powerful and an integral part of ARG dissemination.

In a study performed by Zhao et al. (2022), dogs shared 70% of their ARGs with their owners; the owners shared 72% of their ARGs with their dogs (the largest proportion was represented by genes responsible for resistance against macrolides, aminoglycosides and trimethoprim (*tet(Q)*, *tet(A)*, *mef(A)*, *erm(B)*, *erm(F) and lnu(C)*) [75]. This data shows that the intimate connection between companion animals and their owners is a risk factor for ARG transfer, especially from bacteria residing on the skin and mucosal surfaces [76]. While factors such as interspecies barriers may limit transmission, it has been reported that the shift to cohabitation of domesticated animals with humans has modulated their microbial community [77]. With this, new opportunities were created for the interspecies transfer of ARGs. This is important, as antimicrobial use in small animals has been identified as one of the factors for colonization or infection with ARMs [78]. One of the most critical bacteria are *Enterobacteriaceae—*they are possibly the key drivers of dissemination. These bacteria are present in higher abundances in the dog gut, which enables elevated rates of gene transfer, again proving the concerning role of companion animals as reservoirs of ARGs [79,80]. Further research is needed to determine the causality of the directionality of ARG spread between owners and their companion animals, as well as ways that they can be transferred. A group of especially dangerous and resistant bacteria were reported, ESKAPE, that are particularly hard to treat due to their ability to form biofilms and their resistance against multiple antibiotic classes [81]. The name is an acronym from the first letters of the following genus names: Enterococcus faecium, Staphylococcus aureus, Klebsiella pneumoniae, Actinobacter baumannii, Pseudomonas aeruginosa, Enterobacter spp. [82]. In this group we can find some of the major ARGs that may directly or indirectly affect human health: extended-spectrum beta-lactamase (ESBL) Gram-negative bacteria, methicillin-resistant Staphylococcus aureus (MRSA), and vancomycin-resistant Enterococci (VRE) [79].

Studies show evidence of gene exchange between these bacteria between humans and animals: colonization with MRSA is two times more likely in dog owners when compared to pet-free houses [83]; VRE of dogs have the same genetic lineages as bacteria from patients with hospital-acquired infections [79]; the presence of identical clones of *E.coli* (such as ST131, ST405, ST648) in humans and a number of non-human species and food [79,84,85]; strains of carbapenem-resistant *Actinobacter baumannii* belonging to humans causing urinary tract infection in a cat [86]; and *Klebsiella pneumoniae* from an ST11 human epidemic clone being isolated from dogs and cats in Spain [87]. The available evidence shows that resistant bacteria are shared between animals and humans, that gene transfer occurs between AAB and HAB, and most importantly, that there is the potential to harbor and spread ARGs between microbiomes of different species.

One of the most important factors affecting the dissemination of ARGs is the improper use of antibiotics. However, they are not the only selective pressure contributing to the spread of ARGs [10]. Many substances, such as various non-antibiotic drugs (such as ibuprofen, acetaminophen, and triclosan) or disinfection byproducts (DBPs), have antibiotic-like effects: they cause a significant increase in the expression of stress response genes, as well as DNA damage and repair genes. These processes in turn affect the frequency of mutation, cellular SOS, and oxidative stress response, which enhances the spread of resistance [10]. One of the most commonly used methods for disinfection, chlorination, generates various DBPs such as iodoacetic acid and chlorite bromoacetic acid. These substances exhibit antibiotic-like effects. This means that although the total microbial number decreases after the disinfection, the percentage of antibiotic-resistant bacteria (ARB) can increase, which, in turn, thanks to changes in microbial structure, allows for the dissemination of ARGs [10,88]. This is extremely important in veterinary clinics as the use of disinfectants between patients is a common occurrence and can lead to a higher abundance of ARBs.

Not all substances, however, act in this manner. Microplastics can lead to enhanced spread of ARGs through different mechanisms: the direct absorption of ARGs (the rough, fixed surface is favored for conjugation) [89]; the provision of a surface for microbial colonization (more bacteria promotes HGT) [10]; the transport of ARGs from and to distant places (thanks to the high durability of plastics and their ubiquity) [90]; and an increase in bacteria cell permeability (better propagation) [91]. Many metal nanoparticles and nano metal oxides can enhance HGT via conjugation and transformation by damaging and/or altering cell permeability directly. Metal ions such as Zn^2+^ or Ag^+^ can cause oxidative stress, leading to the promotion of ARG spread [10,92]. Similarly to metal oxide and microplastics, inorganic chemicals (ammonia, selenate, carbon dioxide) are able to damage bacterial cells, leading to increased permeability and transfer efficiency for conjugation and transformation [10,93]. Carbon dioxide can also improve a bacterial cell’s hydrophobicity and change the cell surface, both of which benefit cell-to-cell contact. Additionally, by releasing Ca^2+^, it provides the bacteria with power for DNA uptake [94]. Other chemicals such as artificial sweeteners (aspartame, sucralose, saccharin), preservatives (malachite green, trioxymethylene), or herbicides (glyphosate, microcystins) also play a role in ARG dissemination through the aforementioned mechanisms. Different types of substances exhibit similar effects on the spread of antimicrobial resistance, but the mechanisms, components, and their relation to bacteria and each other vary greatly, making the fight against ARGs so much more difficult [10]. Additionally, the conditions that these processes take place in also matter significantly. These conditions are not limited to the most known and routinely quoted examples such as pH, temperature, humidity, or pressure. It has been documented that electric and sound fields stimulate spread through ways which are not necessarily obvious [95]. Ultrasound (with an intensity below 0.05 W/cm^2^) causes this thanks to the acoustic cavitation effect, which triggers sonoporation (the creation of transient pores in a cell membrane), raises the contact rate between bacteria, and up-regulates the expression of conjunction genes [96]. This only proves that ARGs’ global dissemination is not a problem caused solely by antibiotics and is so much more complex than we ever thought. It is a problem that is not limited to only one field of science, as it draws its complexity from all of physics and chemistry [10].

With the introduction of antibiotics in the late 1930s and 1940s (sulfonamides and penicillin) came acceleration in the development of resistance to said antibiotics [81]. Several years before the widespread availability of penicillin, an experiment was performed and bacterial penicillinases were identified [81]. There are a large number of methods by which bacteria evade or resist antibiotics. For example, despite introducing new Beta-lactams, bacteria regularly and quickly evolve, rendering them almost useless. The estimate is that there are over 1000 known Beta-lactamases (the enzyme which destroys Beta-lactams) and the list is still growing [81]. One of the most persistent and problematic forms of resistance lies in bacteria’s ability to form biofilms. Not only are they resistant, both physically and chemically, to many conventional antibiotics, they are also incredibly hard to remove and can be troublesome or even life-threatening to many patients [81,97]. Biofilm formation is important in surgical procedures where sterile conditions are key. This problem is particularly prevalent in surgical implants as 65% of healthcare bacterial infections are caused by biofilm-related implant infections (BRIIs) [98]. In veterinary medicine, the bacteria most commonly isolated from dogs and cats in surgical site infections (SSIs; *S. pseudintermedius*, *S. aureus*, *P. aeruginosa*) all have the ability to form biofilms; when combined with a more challenging preoperative preparation of the patient, this presents great potential for BRIIs [99].

## 5. Clinical Significance of the Spread of Resistance Among the Natural Microbiota

**Figure 7:**
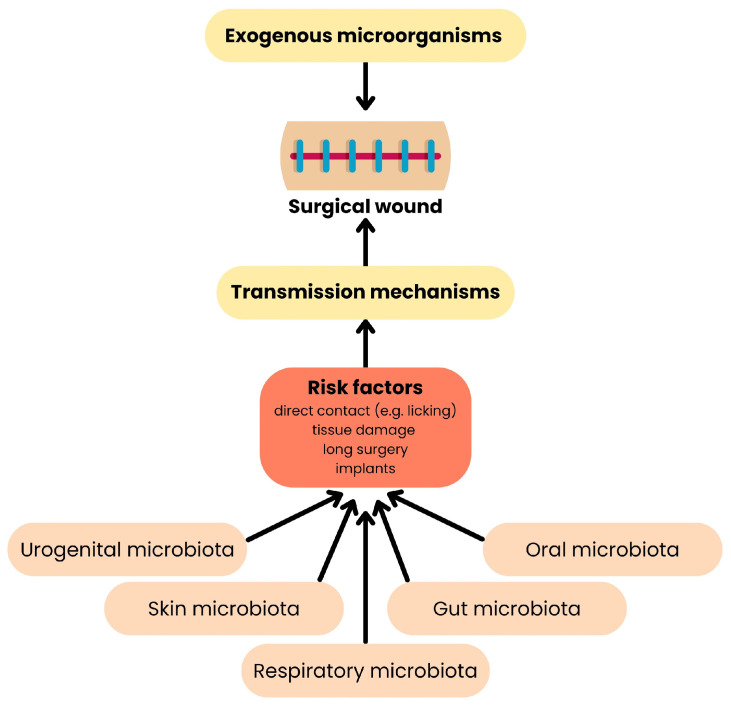
Origin of surgical site infections. This diagram shows how pathogens reach the surgical wound from reservoirs. On the top, exogenous microbes originate in the environment, instruments, or staff. On the bottom, endogenous microbes come from the animal’s own microbiota—skin bacteria or gut microbiota [100].

Postoperative infections in companion animals of endogenous origin represent a significant challenge not only in clinical practice but also in the broader context of public health [100]. Postoperative infections in domestic animals may have either exogenous or endogenous origins, with the latter deriving from the animal’s own natural microbiota and being particularly difficult to predict and eliminate [100] (Figure 7). In recent years, growing attention has been paid to their relevance in the context of public and environmental health, in line with the principles of the One Health approach [101].

A relatively large number of cases of endogenous infections have been described in the literature, which concern both gastrointestinal procedures, orthopedic procedures, and the use of intravenous catheters.

During gastrointestinal procedures, unintentional leakage of intestinal contents into the abdominal cavity can occur, allowing bacteria like *Escherichia coli* to enter the surgical site and cause infection. Risk factors include prolonged surgery duration, the presence of other infections, the animal’s nutritional status, and the use of immunosuppressive drugs [102].

A retrospective study analyzing postoperative wound infections in 276 animals, including 210 dogs and 66 cats following gastrointestinal surgeries, revealed the presence of bacteria such as *Escherichia coli*, part of the natural gut biota, indicating an endogenous source of infection [102,103].

Although orthopedic procedures are usually classified as “clean” surgical procedures, endogenous infections are also reported in this case. A significant source of infection is intestinal biota, especially *Escherichia coli*. It can enter the surgical site through unhygienic behavior of the animal (e.g., licking the wound), preoperative contamination, or improper preparation of the surgical site [103]. The risk of endogenous infections also increases in the case of long surgical procedures, especially those lasting more than 90 min, and in situations where an implant is present in the wound [104]. Additionally, risk factors may include shaving the surgical site (which damages the epidermis) or the presence of comorbidities (e.g., diabetes) [103].

The literature reports a case of postoperative wound infection in a dog following lumbar spine decompression, from which *Salmonella enterica* serotype Dublin was isolated, suggesting an endogenous source [105]. According to the authors hypothesis, the infection likely stemmed from the ingestion of contaminated raw food, leading to gastrointestinal colonization, which led to infection. The article does not directly provide the route of infection, but it can be assumed that the wound was contaminated by the dog licking it, or by insufficient preparation of the surgical site. Contributing factors included the consumption of raw food, immunosuppressive therapy, and concurrent infections.

A separate retrospective study conducted between 2021 and 2023 examines SSIs following orthopedic surgery in dogs and cats. The most frequently isolated bacterium was *Staphylococcus pseudintermedius* (29.78% of all isolates), originating from the natural skin microbiota. The study includes cases involving both open and closed fractures, demonstrating that despite aseptic protocols, skin bacteria can enter the surgical wound. Infections occurred primarily in open fractures and in the presence of implants, with factors such as immunosuppression and foreign bodies increasing the risk [106].

In a study by Crisi et al. (2022), 116 patients, including 76 dogs and 40 cats, who had peripheral intravenous catheters (PIVCs) inserted in a hospital setting, were analyzed [99]. The aim was to determine the incidence of complications, including infections. After catheter removal, 20.7% of cases had a positive culture result, indicating the presence of infection. The most frequently isolated microorganisms included *Staphylococcus intermedius*, *Escherichia coli*, *Pseudomonas aeruginosa*, and *Enterobacter* spp.—bacteria typical of the endogenous microbiota of the skin and gastrointestinal tract. The main risk factor for the development of infection was the duration of catheter insertion—the longer it remained in place, the higher the risk of colonization and infection [99].

Perioperative procedures in dogs and cats also carry a risk of endogenous infections, primarily due to the disruption of natural barriers such as the skin and mucous membranes. The introduction of foreign bodies, such as intravenous or urinary catheters, further increases this risk. Additional contributing factors include prolonged catheterization, mucosal trauma during surgical or diagnostic procedures, and micro-injuries caused by preoperative shaving (Table 3) [99,103,106].

In the event of a breach of anatomical barriers, particularly in the presence of a wound, specific protocols should be implemented to prevent infection. Depending on the type of procedure performed, wounds are classified as clean, clean-contaminated, contaminated, or dirty, and depending on this, there are recommendations for rational perioperative antibiotic therapy (Table 4) [115].

Gastrointestinal and hepatobiliary surgeries are considered clean-contaminated procedures [116]. This classification is supported by retrospective studies showing postoperative infections caused by *Escherichia coli*, a normal commensal of the gut microbiota [117]. Recommendations for the use of antibiotics in this case are presented in Table 3. In contrast, contaminated wounds include traumatic injuries, breaches in sterile technique, or acute inflammation [115]. Orthopedic cases involving open fractures, with infections caused by skin commensals such as *Staphylococcus pseudintermedius*, require therapeutic antibiotic treatment, highlighting the importance of selecting appropriate antibiotics and closely monitoring the patient [106]. Dirty wounds, on the other hand, are characterized by the presence of clinical infection, necrotic tissue, or foreign bodies [115]. Conditions such as primary peritonitis or infections linked to intravenous catheter use fall into this category. Management of dirty wounds necessitates intensive antibiotic therapy guided by culture and sensitivity testing to effectively control the infection.

All of these cases share a common pattern: the source of infection was the animal’s own microbiota, including organisms such as *Escherichia coli*, *Staphylococcus pseudintermedius*, and *Salmonella*, which are normally harmless commensals. Under conditions such as surgery or immunosuppression, these endogenous organisms can become opportunistic pathogens [118]. Within the One Health framework, this highlights the importance of monitoring and managing the microbiome not only in humans but also in animals, particularly in hospital and veterinary environments where cross-species transmission of pathogens is possible [119]. The frequent use of antibiotics to treat such infections, especially when caused by resistant strains, can lead to treatment failures and prolong recovery, further contributing to the global issue of antimicrobial resistance [120]. Many of these infections occurred in clinical settings—during surgery or in the presence of catheters or implants. This underlines the need for rigorous hygiene and infection control standards in veterinary clinics, on par with those in human healthcare, to prevent pathogen spread between animals and humans [121]. Environmental and dietary factors also play a role, as evidenced by the *Escherichia coli* and *Salmonella* case linked to a raw meat diet (BARF—Biologically Appropriate Raw Food) [122]. Such feeding practices create an epidemiological pathway in the One Health chain—food to animal to human—posing a zoonotic risk.

Endogenous infections in companion animals, particularly in clinical settings, represent a transboundary health challenge. Addressing this issue requires joint efforts between veterinarians and medical professionals in the areas of prevention and antibiotic treatment. The One Health approach makes it clear that shared bacteria, shared environments, and shared antibiotic use mean the health of humans and animals cannot be considered in isolation [123]. Coordinated antimicrobial policies, microbiome and resistance monitoring in companion animals, owner education on nutrition and hygiene, and high sanitary standards in veterinary care are essential to address this growing health challenge.

## 6. The Importance of Animal Microbiota for Human Health

Changing attitudes towards animals (especially dogs and cats), as well as their increasingly close interactions with humans, may increase the risk of zoonoses, including the transmission of AMR [124]. The growing interest in this phenomenon, probably related to the increased awareness of One Health aspects, shows that such transmission may negatively affect human health. Examples of drugs, along with the bacteria they target, as well as the genes and mechanism of transfer of resistance, are presented in the table (Table 5).

Bacteria present in the oral cavity, intestines, or on the skin of dogs and cats may contain plasmids with ARGs. They can be transferred to bacteria pathogenic to humans, e.g., through contact with saliva, fur, feces, or scratches, which can lead to the development of AMR and difficulties in treating certain diseases [124]. HGT may lead to the development of more virulent strains that can infect humans more quickly and effectively [124].

The analysis of ARGs in humans, dogs, and cats presented in table (Table 6) reveals significant similarities in the types of genes identified, their anatomical distribution, and the potential for interspecies transmission. Notably, genes such as *bla*_CTX-M_ (extended-spectrum β-lactamases—ESBL), *mec*A (methicillin resistance—MRSA/MRSP), and *qnr*S/*qnr*B (fluoroquinolone resistance) are detected across all three host species, frequently localized in the same anatomical sites, including the gastrointestinal tract, skin, nasal cavity, and urinary system. The concurrent presence of identical ARGs in the same anatomical niches of humans and companion animals—particularly the intestinal tract—suggests that the gut microbiota acts as a major reservoir and convergence point for antimicrobial resistance. The gastrointestinal tract exhibits the highest diversity of ARGs. Horizontal gene transfer mechanisms, including plasmids and class 1 integrons, contribute to the dissemination of these resistance determinants within and between host species. Additionally, the skin, oral cavity, and nasal passages represent important sites for colonization by resistant strains, especially staphylococci (*Staphylococcus pseudintermedius*, *Staphylococcus aureus*). The detection of genes such as *mecA* and *blaZ* in these locations facilitates contact-based transmission via direct physical interaction, shared surfaces, or close proximity between humans and animals. Dogs have been identified as more frequent carriers of resistant bacteria compared to cats; however, both species harbor most of the clinically relevant resistance genes. The zoonotic potential of cats, although less documented, remains a concern, particularly in immunocompromised environments. Reports of clonal similarity between ESBL-producing *E. coli* and MRSP isolates from dogs and their owners further support the possibility of interspecies bacterial exchange within household settings. These findings underscore that humans may act both as sources and recipients of resistant bacteria associated with companion animals, posing a substantial challenge within the One Health framework.

The problem of AMR is the result of interactions between human, animal, and environmental health. AMR develops in high-density environments, such as medical facilities or animal farms, where the widespread use of antibiotics promotes the proliferation of resistant bacteria [35]. Common behaviors, such as sleeping with an animal, allowing face licking, and sharing objects, can also increase the risk of bacterial transfer between humans and animals [147]. Moreover, antibiotic-induced dysbiosis can alter companion-animal behavior via the gut–brain axis—through changes in microbially derived neurotransmitters like GABA, dopamine, and serotonin—potentially modifying close-contact interactions that facilitate resistance-gene exchange [148]. There is no lack of reports of clinical examples of bacterial transfer and resistance determinants between humans and their animals in the literature. Studies have shown that pets, especially those in close contact with their owners, can be a source of *Pasteurella multocida*. An example is the case described by Chomel and Sun (2011), in which a patient with sinusitis had close contact with a cat that licked her daily [147]. The biochemical and genotypic patterns of bacteria from nasal swabs from the patient and the cat’s saliva were very similar, suggesting direct transfer of bacteria [147]. Among cases of infection in infants, *P. multocida* infection (including meningitis) has often occurred after animals, such as dogs, licked the infants or had contact with them, for example, by sharing a bed [149].

In Japan, Kikuchi et al. (2004) described a case of staphylococci transfer between human and animal [150]. A 51-year-old woman presented with irritation and a purulent discharge from her right ear. She had previously undergone tympanoplasty and mastoidectomy for chronic otitis media with cholesteatoma. Examination revealed purulent discharge and inflammation in the operated mastoid cavity, without systemic signs of infection. *Staphylococcus intermedius* was isolated. Despite two months of local treatment, symptoms persisted. An interview was conducted, and it was determined that the patient had a dog that sometimes licked her ears. *S. intermedius* was also isolated from samples of the dog’s saliva and skin. Genetic analysis showed that the strains from the patient and from the dog’s oral cavity were identical, confirming the source of infection [150]. A similar case occurred in a 28-year-old woman who developed a methicillin-resistant sinus infection. The patient had close contact with her dog, including frequent kissing of the dog’s face. The dog had pyoderma, a bacterial skin infection, which was treated with antibiotics. Microbiological testing showed that both the *S. intermedius* strain from the patient’s nose and from her dog were identical [151]. In both cases, the bacteria isolated from the patients and their dogs were genotypically identical (PFGE confirmation), suggesting animal-to-human transmission.

Furthermore, studies show that the transmission of AMR can occur both from animals to humans and from humans to animals. This can result in a phenomenon called “reverse zoonosis”, which involves the transfer of bacteria from humans to animals, which can lead to new sources of infection in animal populations [35]. An example may be the case of a 76-year-old man who was diagnosed with cellulitis with substantial erythema in his right bicep muscle. Multidrug-resistant MRSA was isolated from his skin. After treatment with vancomycin, there was improvement, but after some time, symptoms recurred around the ankle. He was found to be carrying MRSA with an identical resistance profile to the previous isolates. At the same time, his dog, previously operated on for a cruciate ligament rupture, was admitted with severe cellulitis of the neck that had not responded to previous treatment with cephalexin. Cultures from the neck wound and knee joint revealed multidrug-resistant MRSA. Despite intensive treatment, the dog’s condition deteriorated (systemic inflammatory response syndrome, skin necrosis, septic shock) and the animal was euthanized. Genetic testing showed that the MRSA in the dog and owner were indistinguishable, indicating interspecies transmission. The probable source of MRSA was a human, and the dog became infected through contact with the owner while having an open post-operative wound, which he had previously torn by chewing on sutures [152]. Information about cases from the literature is presented in Table 7.

According to the One Health concept, the problem of transferring ARGs requires a holistic approach, taking into account all links in this cycle: humans, animals, and the environment. Preventive actions that can be taken by the owner include: regular veterinary examinations of animals, maintaining hygiene after contact with the animal, and avoiding eating and sleeping together [124]. Veterinarians may be required to use antibiotics judiciously to minimize the risk of AMR, as well as to educate owners about the risks [32]. Preventive measures to limit the spread of ARGs are presented in the table (Table 8).

## 7. Conclusions

The growing understanding of the natural microbiota of dogs and cats underscores its potential role as both a reservoir and vector of ARGs. Even in healthy companion animals, commensal bacterial populations—particularly those inhabiting the skin, oral cavity, gastrointestinal tract, and respiratory system—can harbor ARGs, especially under selective pressures such as prior antibiotic exposure. Within this microbial ecosystem, horizontal gene transfer mechanisms such as conjugation, transduction, and transformation facilitate the dissemination of resistance determinants both within and between bacterial species.

Close, everyday interactions between humans and their pets—including licking, co-sleeping, and shared living environments—intensify the risk of cross-species transmission. Documented cases involving methicillin-resistant *Staphylococcus pseudintermedius* (MRSP), MRSA, and extended-spectrum β-lactamase (ESBL)-producing *Escherichia coli* illustrate that zoonotic and anthroponotic exchanges of resistant bacteria are not only possible but increasingly observed.

In the One Health context, this highlights the complexity and urgency of addressing AMR at the human–animal-environment interface. Recognizing that the commensal microbiota of dogs and cats may contribute to the community-level ARG pool, coordinated action becomes essential. Effective strategies must include routine surveillance, prudent antimicrobial use in veterinary medicine, improved hygiene practices, and public awareness initiatives. Only integrated efforts across veterinary, medical, environmental, and public health disciplines can offer hope to mitigate the spread of AMR and safeguard antimicrobial efficacy for both human and animal populations. 

## Figures and Tables

**Table 1:** Composition of the skin microbiota [38].

Phylum	Species Found in Dogs	Species Found in Cats
Proteobacteria	*Capnocytophaga canimorsus*, *C. canis*, *Paracoccus marcusii*, *Sphingomonas aerolata*	(less frequently identified at species level)
Bacteroidota	*Porphyromonas gulae*, *P. canoris*	-
Actinobacteria	*Cutibacterium* spp.	*Cutibacterium* spp.
Firmicutes	*Staphylococcus pseudintermedius*, *Streptococcus canis*	*Staphylococcus felis* and other *Staph.* spp., *Streptococcus* spp.

**Table 2:** Overview of nasal and oral microbiota in dogs and cats—including anatomical niches, breed differences, and clinical/zoonotic relevance [27,29,30,36,37,38,39,40,41,43,45,46,47].

System	Species/Type	Breed/Skull Type	Anatomical Niche	Dominant Bacteria (Phyla/Genera)	Clinical/Zoonotic Significance	Citations
Nasal	Dog	Mesocephalic/dolichocephalic	Nasal cavity	↑ *Proteobacteria* ↓ *Firmicutes*, *Actinomycetota*	Typical, balanced microbiota; may contribute to respiratory immune defense	[43,45,46,47]
		Brachycephalic	Nasal cavity	↑ *Firmicutes*, *Actinomycetota* ↓ *Proteobacteria*	Microbiota shifts linked to anatomical traits; higher infection risk	[43,45,46,47]
	Cat	– (no breed-specific data)	Nasal cavity	↑ *Proteobacteria* medium: *Firmicutes* no data: *Actinomycetota*	Similar to dogs; breed-related studies are lacking	[27,30,36,37,38,39,40,41,46,47]
Oral	Dog	Maltese, Teddy (toy breeds)	Saliva	*Porphyromonas*, *Moraxella*	*Porphyromonas*—linked to periodontal disease; *Moraxella*—opportunistic respiratory pathogen; zoonotic potential	[27,29,36,37,38,39,40,41]
		Golden Retriever	Saliva	*Neisseria*, *Streptococcus*	*Streptococcus*—may cause infections in humans; *Neisseria*—some species are pathogenic	[27,29,36,37,38,39,40,41]
	Cat	British Shorthair, Ragdoll	Saliva	*Porphyromonas*, *Moraxella*	Similar to toy dog breeds; risk of periodontal infections and bacterial transmission	[27,30,36,37,38,39,40,41]
		Chinese garden cat	Saliva	*Porphyromonas*, *Fusobacterium*	*Fusobacterium*—anaerobes associated with inflammation and oral infections	[27,30,36,37,38,39,40,41]
	Dog and cat	-	Supragingival plaque	*Proteobacteria*, *Bacteroidota*, *Firmicutes*, ↑ *Actinobacteria*, *Saccharibacteria*	Highest bacterial diversity; key site for periodontal disease	[27,29,30,36,37,38,39,40,41]
		-	Buccal mucosa	*Proteobacteria*, *Firmicutes*	Less diverse; relatively stable and less pathogenic flora	[27,29,30,36,37,38,39,40,41]
		-	Dorsum of tongue	*Bacteroidota*, *Firmicutes*	Anaerobes may thrive; possible source of halitosis	[27,29,30,36,37,38,39,40,41]
		-	Saliva	*Proteobacteria*, *Bacteroidota*, *Firmicutes*	Breed-dependent differences; influences oral and general health, as well as zoonotic risk	[27,29,30,36,37,38,39,40,41]

↑—increased abundance/dominance of the given bacterial type; ↓—decreased abundance/reduced presence of the bacterial type.

**Table 3:** Risks and preventive measures related to medical procedures [100,107,108,109,110,111,112,113,114].

Procedure	Risk Description	Preventive Recommendations	Citations
Use of Intravenous Catheters (especially in the cervical region)	Prolonged catheter use, particularly in the neck area, can cause local infections, external jugular vein thrombosis, and bacteremia, potentially leading to spontaneous bacterial peritonitis (SBP).	Maintain strict aseptic technique; regularly monitor catheter sites; avoid prolonged use when possible	[107,109]
Endoscopic procedures	May cause transient bacteremia due to mucosal damage and bacterial translocation from the oral or gastrointestinal tract into the bloodstream.	Ensure aseptic conditions during procedures; consider antibiotic prophylaxis in high-risk cases.	[107]
Paracentesis (abdominal fluid aspiration)	Risk of introducing bacteria into the abdominal cavity, especially if performed non-aseptically; bacteremia may lead to SBP.	Perform under aseptic conditions; monitor patients closely after the procedure.	[107,108]
Use of peritoneal dialysis catheters	Can introduce bacterial infections, especially with poor hygiene; contact with pets may be a source of zoonotic transmission.	Ensure strict hygiene and aseptic handling; limit contact with potential infection sources (e.g., other animals or humans with infections).	[107,108]
Urological procedures (e.g., bladder catheterization)	Can lead to urinary tract infections and bacteremia, which may result in SBP.	Maintain aseptic technique during catheterization; minimize catheter duration; monitor for signs of infection.	[107]
Preoperative shaving	Can cause microtraumas, which become entry points for bacteria, increasing the risk of SSIs.	Avoid shaving with razors; prefer clipping or depilatory creams; perform hair removal immediately before surgery.	[111,112,113]
Use of razors for shaving	Causes epidermal damage, increasing the likelihood of SSIs.	Use safer hair removal methods (e.g., clippers); clipping of the surgical site <4 h prior to the procedure; avoid using razors perioperatively.	[112]
Skin infections as a source of endogenous infection	Naturally occurring skin bacteria may cause infections if the skin is damaged or contaminated during procedures.	Conduct dermatological evaluations prior to surgery; treat pre-existing skin infections before performing procedures.	[114]

**Table 4:** Recommendations for rational perioperative antibiotic therapy depending on the type of wound [115].

Wound Classification	Clinical Description	Antibiotic Therapy Recommendations
Clean wound	Planned surgical procedure with no entry into the respiratory, gastrointestinal, or genitourinary tract; no infection or trauma.	Antibiotics generally not required, unless the procedure exceeds 90 min or involves implants.
Clean-contaminated wound	Entry into the respiratory, gastrointestinal, or genitourinary tract under controlled conditions without major contamination.	Prophylactic antibiotics recommended, e.g., for cystotomy or gastrotomy.
Contaminated wound	Fresh traumatic wounds, spillage from the gastrointestinal tract, major break in sterile technique, or acute non-purulent inflammation.	Therapeutic antibiotics indicated, especially if infection is suspected before surgery.
Dirty wound	Clinical infection present, necrotic tissue, foreign material, or chronic wound contamination.	Intensive antibiotic therapy required, based on culture and sensitivity results.

**Table 5:** Overview of antimicrobial drug classes, resistance genes, resistance mechanisms, and representative bacterial pathogens [125,126,127,128,129,130,131,132,133,134,135,136].

Drug Class	Drug Example	Resistance Gene(s)	Resistance Mechanism	Bacteria Example	Citations
β-lactams	Ampicillin	*blaTEM*, *blaSHV*	Bacteria produce β-lactamases—enzymes that hydrolyze the β-lactam ring, rendering the antibiotic ineffective.	*E. coli*, *Klebsiella pneumoniae*	[125]
	Cefotaxime	*bla*_CTX-M_	Bacteria produce ESBLs that can hydrolyze a wide range of cephalosporins.	*E. coli*, *Enterobacter* spp.	[125]
	Oxacillin	*mecA*	*mecA* encodes a modified penicillin-binding protein (PBP2a) with low affinity for β-lactams, allowing cell wall synthesis to continue	*Staphylococcus aureus* (MRSA)	[125]
Carbapenems	Imipenem, Meropenem	*blaKPC*, *blaNDM*, *blaVIM*, *blaOXA-48*	Bacteria produce carbapenemases—enzymes capable of hydrolyzing carbapenems, among the most potent β-lactams.	*K. pneumoniae*, *P. aeruginosa*, *A. baumannii*	[127]
Aminoglycosides	Gentamicin	*aac(6′)-Ib*, *aph(3′)*, *ant(2″)*	Bacterial enzymes chemically modify the antibiotic using acetylation or phosphorylation, preventing ribosome binding.	*E. coli*, *P. aeruginosa*, *Enterococcus* spp.	[125,126,128]
Tetracyclines	Doxycycline	*tet(A)*, *tet(B)*, *tet(M)*	Efflux pumps remove the antibiotic from the cell; ribosomal protection proteins prevent drug binding to the ribosome.	*E. coli*, *Streptococcus* spp., *Enterococcus* spp.	[129]
Quinolones	Ciprofloxacin	*qnrA*, *qnrB*, *qnrS*, *aac(6′)-Ib-cr*	Qnr proteins protect DNA gyrase from inhibition; modifying enzymes reduce the antibiotic activity.	*E. coli*, *Salmonella* spp., *Klebsiella* spp.	[126,127]
		Mutations in *gyrA*, *parC*	Mutations alter the structure of DNA gyrase or topoisomerase IV, preventing the antibiotic from binding effectively.	*Campylobacter* spp., *Neisseria gonorrhoeae*	[126,127]
Macrolides	Azithromycin	*erm(B)*, *mef(A/E)*	erm genes encode rRNA methylation (modifying the binding site), and mef genes encode efflux pumps that expel the drug.	*S. pneumoniae*, *S. pyogenes*, *Mycoplasma* spp.	[127,130,136]
Glycopeptides	Vancomycin	*vanA*, *vanB*	The bacteria alter their cell wall precursors from D-Ala-D-Ala to D-Ala-D-Lac, which reduces vancomycin binding.	*E. faecium*, *E. faecalis*	[133]
Sulfonamides	Sulfamethoxazole	*sul1*, *sul2*, *sul3*	The genes encode an alternative dihydropteroate synthase that is not inhibited by the antibiotic—allowing folic acid synthesis to continue.	*E. coli*, *Salmonella* spp., *Shigella* spp.	[125,127,129,131,135]
Chloramphenicol	Chloramphenicol	*cat*, *floR*, *cmlA*	The antibiotic is inactivated by acetylation, and efflux pumps remove it from the cell.	*Salmonella* spp., *E. coli*, *K. pneumoniae*	[132]
Rifampin	Rifampin	*rpoB* mutations	Mutations in rpoB alter the binding site on RNA polymerase, making it resistant to inhibition.	*Mycobacterium tuberculosis*, *S. aureus*	[125,134]

**Table 6:** Comparative distribution of ARGs in humans, dogs, and cats by anatomical site [32,137,138,139,140,141,142,143,144,145,146].

ARG	Antibiotic Resistance	Colonized Species	Typical Anatomical Locations (Dog/Cat/Human)	Typical Microbiota	Notes	Citations
*bla*_CTX-M_	ESBL —cephalosporins	Dog: yes (frequent)	Dog: intestines, urinary tract	*E. coli*, *Enterobacteriaceae*	Plasmid-mediated; widespread. ST131 in humans genetically related to canine isolates.	[32,138,139,140]
		Cat: yes (less common)	Cat: intestines			
		Human: yes (frequent)	Human: intestines, urinary tract			
*bla*TEM, *bla*SHV	Penicillins, early cephalosporins	Dog: yes	Dog: intestines	*Enterobacteriaceae*	Common in both commensal and pathogenic strains.	[32,138,139,140,146]
		Cat: yes	Cat: intestines			
		Human: yes	Human: intestines			
*mecA*	Methicillin —β-lactams	Dog: yes	Dog: skin, nose	*S. pseudintermedius*, *S. aureus*	Zoonotic transfer risk; MRSP/MRSA detected across species.	[32,138,139,141,142,146]
		Cat: less common	Cat: skin, nose			
		Human: yes (carrier state)	Human: nose, skin			
*bla*Z	Penicillins	Dog: very common	Dog: skin, nose	*Staphylococcus* spp.	Highly prevalent in canine staphylococci.	[32,138,139,146]
		Cat: yes	Cat: skin, nose			
		Human: yes	Human: skin, nose			
*tet(M)*, *tet(A)*, *tet(B)*	Tetracyclines	Dog: yes	Dog: oral cavity, intestines, nose	*Porphyromonas*, *Fusobacterium*, *E. coli*	Frequently associated with mobile genetic elements; found in biofilms.	[32,137,138,139,146]
		Cat: yes	Cat: oral cavity, intestines			
		Human: yes	Human: oral cavity, intestines			
*erm(B)*, *erm(C)*	Macrolides, lincosamides	Dog: yes	Dog: oral cavity, intestines, nose	*Enterococcus*, *Staphylococcus*, anaerobes	Broadly present in multiple genera, especially anaerobes and staphylococci.	[32,137,138,139,143,145,146]
		Cat: yes	Cat: oral cavity, intestines			
		Human: yes	Human: oral cavity, intestines, nose			
*aac(6′)-Ib*, *aph(3′)-IIIa*	Aminoglycosides	Dog: yes	Dog: intestines, nose	*E. coli*, *Enterococcus*	Found in multidrug-resistant isolates from animals and humans.	[32,138,139,144,146]
		Cat: less common	Cat: intestines			
		Human: yes	Human: intestines, nose			
*qnr* genes (*qnrS*, *qnrB*)	Fluoroquinolones	Dog: yes	Dog: urinary tract, intestines	*Enterobacteriaceae*	Plasmid-mediated quinolone resistance; detected across species.	[32,138,139,144,145,146]
		Cat: yes	Cat: urinary tract, intestines			
		Human: yes	Human: intestines, urine			
*sul1*, *sul2*	Sulfonamides	Dog: yes	Dog: intestines, urine	*E. coli*, *Bacteroides*	Commonly associated with class 1 integrons; easily disseminated.	[32,138,139]
		Cat: yes	Cat: intestines, urine			
		Human: yes	Human: intestines, urine			
*dfrA*	Trimethoprim	Dog: yes	Dog: intestines	*Enterococcus*, *E. coli*	Often co-selected with *sul* genes (*sul1*, *sul2*).	[32,138,139]
		Cat: yes	Cat: intestines			
		Human: yes	Human: intestines			

**Table 7:** Cases of animal-to-human bacterial transfer [147,149,150,151,152].

Bacteria	Direction of Transfer	Host(s)	Details	Citations
*Pasteurella multocida*	Cat → Human	Cat and woman with sinusitis	Woman had daily contact with a cat that licked her; nasal and saliva swabs showed biochemically and genotypically similar strains.	[147]
	Dog → Infant	Dogs and infants	Infants developed infections, including meningitis, after dogs licked them or shared beds.	[149]
*Staphylococcus intermedius*	Dog → Human	Dog and 51-year-old woman with ear infection	Dog licked patient’s ears; identical bacterial strains isolated from dog’s saliva and patient’s ear.	[150]
*Staphylococcus intermedius* (MR strain)	Dog → Human	Dog with pyoderma and 28-year-old woman with methicillin-resistant sinusitis	Frequent face contact; bacterial strains from dog’s skin and woman’s nose were identical.	[151]
Methicillin-resistant *Staphylococcus aureus*	Human → Dog, then Dog → Human	76-year-old man and his dog with severe cellulitis after surgery	Man had recurring MRSA; dog, recovering from surgery, became infected. Genetic testing confirmed identical multidrug-resistant strains in both, suggesting bidirectional transmission.	[152]

**Table 8:** Preventive measures to limit the spread of ARGs in the One Health context [153,154,155,156].

	At the Owner Level	At the Veterinary Level	Citations	
			Owner	Veterinarian
Antibiotic use	Regular preventive veterinary check-ups should ensure routine health monitoring, and vaccination helps to reduce the incidence of infections and the need for antibiotic use.	Antibiotic therapy should be based on microbiological diagnostics; antibiotic treatment should be applied only following culture and susceptibility testing.	[153,154,156]	[154,156]
	Improper disposal of antibiotics should be avoided; unused antibiotics should be returned to designated disposal points (e.g., pharmacies or clinics).	Critically important antibiotics should be prescribed only when strictly necessary.	[154,156]	[154,156]
		Veterinarians should educate owners; communication strategies should be implemented to inform owners about the risks of inappropriate antibiotic use.		[153,154]
Preventive measures, biosecurity	Close physical contact should be limited; sharing beds, food, or allowing animals to lick the face should be avoided to minimize the risk of transmission of resistant bacteria.	Biosecurity protocols should be implemented in veterinary settings; infected animals should be isolated, and disinfection procedures should be strictly followed.	[155,156]	[156]
	Hand hygiene should be practiced after animal contact; handwashing is recommended after handling, feeding, or cleaning up after animals to reduce the risk of microbial transmission.		[155,156]	
	The domestic environment should be regularly cleaned; food and water bowls, bedding, and litter boxes should be disinfected regularly, particularly during illness.	Participation in training and awareness programs should be encouraged; continuous education on responsible antibiotic use should be promoted among veterinary professionals.	[153,155,156]	[153,156]

## Data Availability

No new data were created or analyzed in this study. Data sharing is not applicable to this article.

## Abbreviations

The following abbreviations are used in this manuscript:

AAB	Animal-associated bacteria
ARB	Antibiotic-resistant bacteria
ARGs	Antibiotic resistance genes
AMR	Antimicrobial Resistance
BRII	Biofilm-related implant infections
BARF	Biologically Appropriate Raw Food
DBPs	Disinfection byproducts
DI	Dysbiosis index
ESBL-producing *E. coli*	Extended-spectrum β-lactamase-producing *E.coli*
HAB	Human-associated bacteria
HGT	Horizontal gene transfer
MGEs	Mobile genetic elements
MRSA	Methicillin-resistant *S. aureus*
OMVs	Outer membrane vehicles
oriT	Origin of transfer
PIVC	Peripheral intravenous catheters
RAUS	Rational Antibiotic Use System
SBP	Spontaneous bacterial peritonitis
SCFAs	Short-chain fatty acids
SSIs	Surgical site infections
VGT	Vertical gene transfer
VRE	Vancomycin-resistant *Enterococci*
